# Direct-access to sonographic diagnosis of deep vein thrombosis in general practice: a descriptive cohort study

**DOI:** 10.1186/s12875-020-01267-x

**Published:** 2020-09-21

**Authors:** Charlotte W. Appel, Annette M. Balle, Mads M. Krintel, Axel Vittrup, Agnete H. Nielsen, Peter Vedsted

**Affiliations:** 1 Diagnostic Centre, University Research Clinic for Innovative Patient Pathways, Silkeborg Regional Hospital, Silkeborg, Denmark; 2 Department of Clinical Medicine, Aarhus University, Aarhus, Denmark; 3 Research Unit for General Practice, Department of Public Health, Aarhus University, Aarhus, Denmark

**Keywords:** General practice, Denmark, Venous thrombosis, Ultrasonography, Diagnosis, Clinical decision-making

## Abstract

**Background:**

Suspicion of deep vein thrombosis (DVT) is common and requires urgent and efficient investigation due to hazardous prognosis. The traditional diagnostic pathway can be complex and time-consuming, and innovative solutions may provide easy access to diagnostics and better use of healthcare resources. We aimed to describe use, clinical outcomes and time used when providing general practitioners (GPs) with a direct-access pathway to hospital-based, single whole-leg compression ultrasound (CUS) for patients with suspected DVT. Furthermore we aimed to describe the resources used in the new direct-access pathway and compare it with the previous pathway.

**Methods:**

We conducted a 2-year descriptive cohort study (2016–2017) including 449 consecutively referred patients for diagnosis of DVT in a Danish regional hospital. The previous pathway included pre-test at the medical department, a proximal leg CUS if required based on the pre-test and a re-scan if the first CUS was negative. The new pathway included two strategies: 1) a ‘yes-no strategy’, where GPs referred patients directly to whole-leg CUS and if positive, treated at the medical department and if negative, discharged to the GP, 2) a ‘follow-up strategy’ where GPs could require that patients were seen at the medical department, irrespective of the CUS result. Data included extractions from the Radiology Information and Patient Administrative Systems, and mean salaries of healthcare professionals at Silkeborg Regional Hospital, Denmark. Descriptive statistics were used to describe prevalence, timelines and costs.

**Results:**

GPs referred 318 (71%) patients through the yes-no strategy and 131 (29%) via the follow-up strategy with DVT diagnosed in 48 (15%) and 51 (39%) patients, respectively (*p* < 0.001). For the 263 patients completed after CUS in the yes-no strategy, median pathway time was 24 min (IQI: 16–36), and for those with DVT (including both strategies) 202 min (IQI: 158–273). Direct-access pathway costs were €49.7 less per patient than the previous pathway.

**Conclusion:**

Direct-access to CUS for suspected DVT was achievable, had short time intervals and required fewer resources. The difference in DVT prevalence indicates that GPs distinguish between patients with low and high risk of DVT.

## Background

Lower extremity deep vein thrombosis (DVT) is a common disease with an annual incidence of one in 1000 adults increasing with age [1, 2]. DVT can lead to potentially life-threatening complications such as pulmonary embolism or trigger the development of post-thrombotic syndrome [2, 3]. Accordingly, fast, efficient diagnosis and treatment are essential [4].

Traditionally, the diagnostic tool used to diagnose DVT is compression ultrasound (CUS) of the proximal leg vein where about 20% will have a DVT [5]. The cost and number of negative tests has led to strategies used to triage patients with suspected DVT, in order to rule out DVT and avoid CUS using pre-test probability assessment (i.e. Wells score) and a D-dimer test [4, 6]. When a CUS is required, but proves to be negative, and the D-Dimer is positive, CUS is repeated 6–8 days later to assess for an undetected distal DVT [5]. Although the probability assessments allow avoiding CUS in 20–30% of outpatients [7, 8], the diagnostic pathway is complex and time-consuming.

Patients typically present to general practice with nonspecific symptoms including swelling, pain and tenderness [3]. In Denmark, the general practitioner (GP) is the gatekeeper to specialised healthcare, and if DVT is suspected, the patient is referred to the hospital for diagnostics which includes possible involvement and resources from several departments i.e. medical department, radiology department and the laboratory.

To ensure easy, simple and direct access to diagnosis of DVT it is important to develop innovative solutions, which also provide better use of healthcare resources. In the diagnosis of DVT a single whole-leg CUS is found to capture distal DVT and thereby eliminate repeated CUS [9–11]. This opens for the possibility to organise a pathway with direct-access to whole-leg CUS for the GP and same-day investigation for all referred patients without additional testing or repeated CUS.

Based on this, the Regional Hospital Silkeborg in Denmark developed and implemented a new, direct-access pathway to hospital-based, single, whole-leg compression ultrasound for patients where the GPs suspect DVT. We aim to describe the use, clinical outcomes and time when Danish GPs were provided with this new diagnostic pathway. Furthermore, we aimed to describe the resources used in the new direct-access pathway and compare it with the previous pathway.

## Methods

### Design and setting

We conducted a descriptive cohort study on all consecutive patients referred to CUS from GPs during 2016 and 2017. The CUS was performed at Silkeborg Regional Hospital, Denmark, which has a catchment area of 93.000 people and around 50 GPs. The hospital is located in Central Region Denmark, which is one of the five Danish Regions. Other GPs within this region also had the opportunity to refer patients, but they mainly used their local hospital.

### Organisation of direct-access pathway for suspected DVT

**Fig. 1:**
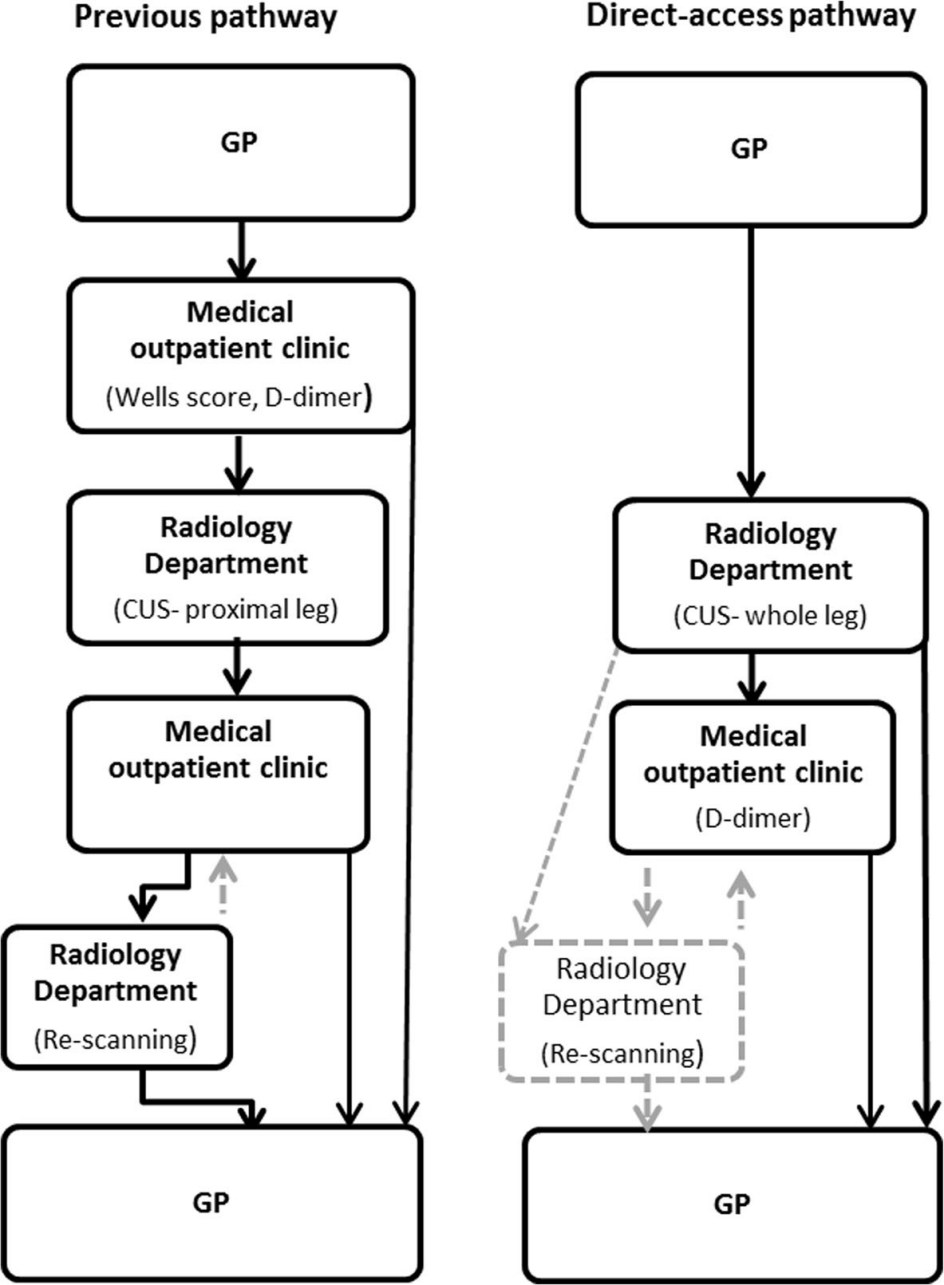
Flowchart of the previous pathway and the new direct-access pathway for patients referred from general practice (GP) under suspicion of lower extremity deep vein thrombosis (DVT)

In October 2015, we designed and implemented a new fast-track pathway, where GPs could refer patients suspected to have DVT directly to a same-day, whole-leg CUS at the Radiology Department (RD), Diagnostic Centre, from 7.30 am to 8.00 pm without a prior D-dimer test (Fig. 1). The indication was the GP’s clinical suspicion, e.g. swelling, pain, redness and warmth. To support the decision to refer a patient the GP was encouraged to use and report the Wells score, but it was not mandatory. The pathway did not include patients with pulmonary symptoms, which raises the suspicion of a pulmonary embolism. In Denmark, these patients are referred directly to critical care procedures.

**Fig. 2:**
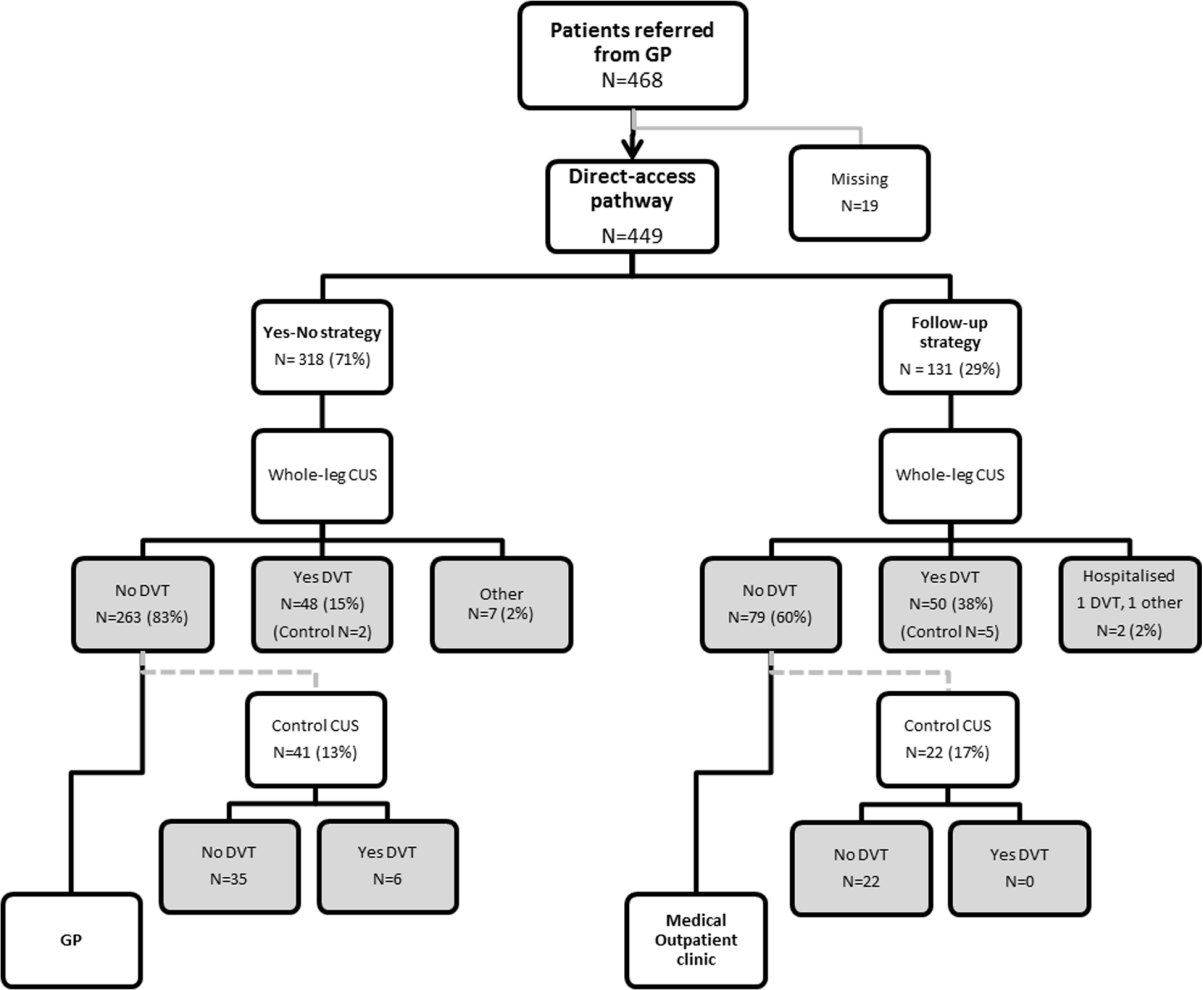
Flowchart of the DVT direct-access pathway for 449 patients referred from general practice (GP) under suspicion of lower extremity deep vein thrombosis (DVT)

The GP could choose between two strategies at referral (Fig. 2); 1) a yes-no strategy, where the referred patient had a whole-leg CUS and, if positive, was sent to the Medical Outpatient Clinic (MOC) for treatment and if negative, sent back to the GP (‘yes-no strategy’), and 2) a follow-up strategy where all referred patients, after whole-leg CUS, were seen in the MOC, irrespective of the CUS result. The latter procedure was used if the GP required a second opinion.

The GPs referred the patient by calling the RD and sending an electronic referral as per usual. The GP received corresponding results electronically through the normal discharge letter. The whole-leg CUS was mainly performed by a certified sonographer, a radiographer with specialised training in CUS of the whole leg. Re-scanning after approximately 1 week was performed only if the first whole-leg CUS was inconclusive.

The previous diagnostic pathway included pre-test assessments for all patients at the MOC (Fig. 1). Patients with an abnormal D-dimer or the combination of a normal D-dimer and an intermediate to high Well’s score went on to a proximal leg CUS. After CUS, all patients were seen at the MOC for treatment if the CUS was positive, or discharged and re-scanned 6–8 days later if negative.

### Study population

All adults (aged 16 years or more) referred by their GP to the new fast-track investigation for DVT at the Diagnostic Centre, Silkeborg Regional Hospital, 1 January 2016–31 December 2017, were identified using the Healthcare Classification System codes from the Radiology Information System (RIS), which keeps records of all radiological investigations. Patients were given a special project code, when referred to the fast-track investigation. New fast-track referrals during a 3-month follow-up period were analysed as events related to the initial CUS, while referrals 3 months apart were classified as a new referral.

### Data

#### Data and time registration

Data on initial diagnose, venous thromboembolic events and death during a 3-month follow-up period after the CUS as well as time-interval registration was extracted from RIS and the Patient Administrative System (PAS), which contains data on all inpatient and outpatient activities in Central Region Denmark. Data included dates and times for referrals, arrivals and examinations (start and end) as well as discharge diagnosis, age, gender, strategy (1 or 2), and radiological investigations. Data was combined using the unique Danish civil registration number assigned to each Dane at birth [12].

### Resources

To estimate differences in resources allocated to different patient pathways for suspected DVT, we estimated the costs if our population had been referred to the previous diagnostic pathway. This was compared with the cost of the new pathway. We assumed that our population would have had the same number of CUS and working hours as patients diagnosed according 2014 guidelines. As suggested by these guidelines, all patients were first seen at the MOC where from approximately 75% were referred to a proximal leg CUS [7]. After CUS, patients were seen at the MOC, and the patients without DVT were re-scanned 6–8 days later. To estimate the overall costs of the two pathways, we first made assumptions on the number of patients going through the different parts of the previous pathway, we used the number of patients going through the different parts of the direct-access pathway, and we estimated the time healthcare professionals used per patient and number of D-dimer. Overall costs were finally estimated based on mean salaries (in EURO (€) [13]) for each group of healthcare professionals at Silkeborg Regional Hospital in 2017 and blood-sample costs.

### Analyses

Descriptive statistics including mean, standard deviations (sd), median and interquartile intervals (IQI) were used for continuous variables and percentages, along with 95% confidence intervals (CI) for categorical variables. Differences between the yes-no strategy and the follow-up strategy were tested with T-test and Chi^2^-test. Patients with missing data were excluded from the analyses. Analyses were conducted using STATA version 15.

## Results

A total of 468 CUS were registered as referrals from general practice to direct-access whole-leg CUS in 2016 and 2017 (Fig. 2). After excluding those with missing dates (*n* = 19), 449 patients were included in the analyses. More than half of the patients were women (57%) and the median age was 65 years (IQI: 52–74) (Table 1). Two patients, both from the follow-up strategy, were hospitalised and excluded from the timeline analysis of the whole pathway (Fig. 2, Table 2).

**Table 1:** Baseline characteristics and clinical findings of patients referred to DVT direct-access pathway^a^ (*N* = 449)

	Direct-access Yes-no strategy (***N*** = 318) ***N*** (%)	Direct-access Follow-up strategy (***N*** = 131) ***N*** (%)	***p***-value	Total (***N*** = 449) ***N*** (%)
Age	64 (50–75)^b^	67 (56–74)^b^	0,026	65 (52–74)^b^
Female	193 (61)	63 (48)	0,014	256 (57)
DVT after initial CUS^a^	48 (15)	51 (39)	< 0.001	99 (22)
Control CUS (N (%))	41 (13)	22 (17)	0,279	63 (14)
DVT after Control CUS	6 (15)	0 (0)	0,065	6 (1)
DVT during follow-up	2 (0.4)	0 (0)	0,363	2 (0.4)
Pulmonary embolism during follow-up	0 (0)	0 (0)	–	0 (0)
Death during follow-up	2 (0.5)	2 (1,5)	0,357	4 (0.9)

^a^*DVT* Deep vein thrombosis, *CUS* Compression ultrasound

^b^Median (IQI)

**Table 2:** Timelines in minutes of DVT direct-access pathway to whole-leg CUS^a^ divided into two strategies

	Direct-access to yes-no			Direct-access with follow-up			All		
	***n***	Mean (sd)	Median (IQI)	***n***	Mean (sd)	Median (IQI)	***n***	Mean (sd)	Median (IQI)
**Time from arrival in RD**^a^ **to CUS**^a^ **start**	318	15.0 (14.9)	11 (4–21)	131	15.3 (16.4)	10 (4–21)	449	15.1 (15.4)	11 (4–21)
**Time for CUS**	318	14.1 (9.0)	12 (9–16)	131	14.4 (8.6)	13 (10–16)	449	14.2 (8.9)	12 (9–16)
**Time for entire pathway - Arrival RD to CUS end**	263	28.5 (18.3)	24 (16–36)	2^b^	–	–	–	–	–
**Time for entire pathway - Arrival RD to MOC**^a^ **end**	55	201.6 (83.0)	204 (147–263)	129	208.2 (92.4)	190 (147–255)	184	206.3 (89.5)	196 (147–256)
**Time for entire pathway** **- DVT-patients**	48	206.6 (82.8)	204 (149–277)	50	222.9 (98.1)	200 (159–268)	98	214.9 (90.9)	202 (158–273)
**Time for pathway** **- All patients**	–	–	–	–	–	–	449	101.5 (105.4)	42 (21–172)

^a^*RD* Radiology Department, *CUS* Compression ultrasound, *MOC* Medical outpatient clinic

^b^Two patients were hospitalised (one with DVT)

Most (71%) were referred through the yes-no strategy (Fig. 2) and in all, 99 (22%) patients received an immediate DVT-diagnosis. In the group referred to the yes-no strategy 15% were diagnosed with DVT, in comparison to 39% in the group referred to the follow-up strategy at MOC (*P* < 0.001).

Of the 449 patients, 63 patients (14%) had a planned re-scan and 6 (10%) of these patients (all within the yes-no-strategy) received a DVT diagnose at that time. Thus, for all referred patients, 105 (23, 95% CI = 19.5 to 27.5) were diagnosed with a DVT.

During the 3-month follow-up, four patients (0.9, 95% CI = 0.24 to 2.27) died. Two of these patients had been diagnosed with DVT, one patient with symptoms related to a known chronic disease and the last patient was further diagnosed with unknown disease. We did not have information on cause of death.

Two patients referred through the yes-no strategy (0.4, 95% CI = 0.1 to 1.6) were diagnosed with DVT during the follow-up period (Table 1). CT pulmonary angiogram was performed in 10 patients on the same day they had their first whole-leg CUS and were diagnosed with DVT, and in 5 patients after the initial whole-leg CUS. No episodes of pulmonary embolisms were registered among the patients during the 3-month follow-up period. Finally, six patients had new referrals through the direct-access pathway (yes-no strategy *n* = 3, follow-up *n* = 3), with no DVTs diagnosed.

All patients had the whole-leg CUS performed on the day of referral. The overall median time from arrival to CUS start was 11 min (IQI: 4–21) (Table 2). The 263 patients referred through the yes-no strategy with no DVT were completed after median 24 min (IQI: 16–36).

For patients referred to the follow-up strategy by their GP, the median time of the entire pathway was 190 min (3.1 h) (IQI: 147–255) (Table 2). For all the 98 patients with an immediate diagnosed DVT, not hospitalised (one patient with DVT was hospitalised), the median time for the entire pathway, including start of treatment, was 202 min (IQI: 158–273). There was no statistically significant difference between the two referral strategies when comparing all time intervals (data not shown).

Table 3 shows the costs of the previous pathway and the direct-access pathway including both strategies. Converted into cost for 449 patients, the direct-access pathway costed €22,307 less than the previous pathway, equivalent to €49.7 less per patient.

**Table 3:** Cost of DVT^a^ direct-access pathway (the yes/no and follow-up strategy combined) and previous pathway

Resources	Previous pathway				Direct-access pathway			
	Time (min)^b^	%^c^	*n*	Costs (€)^d^	Time (min)^b^	%^c^	*n*	Costs (€)^d^
Before CUS^a^								
Medical Outpatient Clinic		100	**449**					
Secretary	2	80	359	342				0
Nurse	30	100	449	7056	–			0
D-Dimer	x	100	449	3920	–			0
Radiology Department		75	**337**			100	**449**	
Secretary	2	80	270	273	3	80	359	545
Radiologist	15	100	337	6280	15	20	90	1677
Radiographer	15	100	337	2535	15	20	90	677
Sonographer	–				15	80	359	2821
Re-scanning		69^e^	232			14	63	
Secretary	2	69	232	235	–			0
Radiologist	15	69	232	4324	–			0
Radiographer	15	69	232	1745	–			0
Sonographer	–				15	14	63	495
After CUS								
Medical Outpatient Clinic		100	**337**			41	**184**	
Secretary	2	100	337	321	2	100	184	176
Nurse	30	100	337	5296	30	100	184	2892
D-Dimer	–				x	100	184	1606
Medical doctor	15	50	169	1907	15	50	92	1038
Total				34,234				11,927

^a^*DVT* Deep vein thrombosis, *CUS* Compression Ultrasound (Previous: proximal leg, Direct-access: whole-leg)

^b^Estimated time used per patient by the different health care professionals

^c^Percent of the population in contact with the health professional or who had D-Dimer taken

^d^Costs (salary and blood sample costs) €

^e^All patients without DVT are re-scanned in previous pathway (DVT *n* = 105)

## Discussion

### Principal findings

Direct-access to whole-leg CUS for suspected DVT provided GPs with a simple, fast-track pathway for suspected DVT. Overall, 23% of those referred had a DVT diagnosis, distributed with a risk of DVT of 15% for the three quarters of patients referred to the yes-no strategy and 39% risk of DVT when referred to a mandatory follow-up at the MOC. Only two patients were diagnosed with a DVT after a negative CUS indicating that the fast-track pathway with the differentiated two routes appeared safe. Sixty percent of all patients were completed with a median time of 24 min without hospitalisation. The direct-access same-day pathway provided an approximately 60% decrease in use of resources in comparison to the previous pathway.

### Strengths and limitations

The study included a well-defined real-life population of patients referred by their GP, and can be generalised to other settings where the GP serves as the first contact. To our best knowledge, this is the first study to evaluate a hospital based DVT pathway focusing on providing GPs with a fast direct access to DVT assessment and enabling the GP to risk-stratify the patient. Furthermore, the hospital based setting ensured specialised treatment without delay or with a second opinion, if necessary.

The strengths of the study include that data were collected prospectively, we had valid data and complete follow-up from the population of patients referred through their GP. However, data based on registries (PAS and RIS) also have limitations. 1) A potential limitation is the use of administrative data on time and date registration. However, we have no reason to expect that time-registration would be systematically under or overestimating the time used, or that the 19 patients excluded from the data set due to missing dates would have changed the results in the study. 2) We did not have data on whether DVT was a proximal or a distal DVT and which treatment was initiated for the DVT, and could therefore not present these data or take into account increased cost of treating distal DVT detected using whole leg CUS. We cannot rule out that the new pathway would cost more if this was included. 3) Often other studies include data on clinical characteristics and risk factors for DVT e.g. pregnancy and previous DVT. The register data did not include these characteristics which makes it difficult to compare the results to other studies, that often exclude patients with these characteristics. The limitations of these register based data could be addressed by manually register the time used for each health professional per patient and by adding data from hospital records. Future studies might include such data. We made assumptions about the time and resources used on detecting DVT based on the previous and present workflow. However, we cannot rule out that the workflow has varied from patient to patient, but we do not expect this to differ in the two periods, why it is considered to be of little importance. Finally, the estimation of resources used does not include all possible cost variables, but it gives an overall picture of the differences in resources used in the previous and the direct-access pathway.

### Findings in relation to other studies

The GPs may have used a ‘wait-and-see’ approach more often in the previous setting. However, the overall risk of DVT when referred through the direct-access pathway was 23%, which is similar to other studies including outpatients with suspected DVT [7, 11, 14]. This indicates that the GPs in our study, despite direct access, did not refer more patients than before. Results concerning the risk of DVT are difficult to compare with other studies, because in these studies, CUS (of the proximal leg) was only performed on patients, who scored high for risk of DVT [7, 11, 14].

Previous studies on DVT pathways have focused on strategies to minimise the number of CUS in general, or whole-leg CUS in particular, by using algorithms including pre-test probability and D-dimer measurements [15]. To our knowledge, no other studies have focused on the timeliness and cost-effectiveness of hospital based pathways for DVT among patients referred by the GP. One newer study has however assessed the diagnostic accuracy of CUS performed by GPs in primary care leaving out pre-test scores and D-dimer. The results suggest that GPs might be an accurate and fast alternative for diagnosis of proximal DVT [16]. Despite these positive results, the sensitivity appeared suboptimal and future studies are needed.

Whole-leg CUS and CUS of the proximal leg (incl. follow-up CUS) are found to be clinically equivalent [11, 15, 17]. Whole-leg CUS has the advantage that a repeated CUS is not necessary to detect if a isolated distal deep vein thrombosis (IDDVT) has extended to proximal veins [9–11]. For most patients this mean, they only have to visit the hospital once. However, routine use of whole-leg CUS as in our study may lead to higher incidence of IDDVT than other diagnostic strategies [17, 18]. There are concerns that detecting IDDVT will lead to over treatment, because IDDVT is generally considered to be more “benign” compared to proximal DVT, and because a standard of care currently does not exist [19]. Previous studies however suggest, that the incidence of later complications such as post thrombotic syndrome, following IDDVT should not be neglected and that surveillance is important [18]. Furthermore, whole-leg CUS enables detection of additional pathology [20, 21]. Thus, performance of the whole-leg investigation in the first instance may lead to improved diagnostic efficiency.

### Implications

Access to fast relevant assessment for DVT is important in general practice. This study shows that a simple same-day access to CUS for suspected DVT appeared safe and efficient. Further, the difference in risk of DVT between the yes-no strategy and the follow-up strategy indicates that the GPs were able to distinguish patients with the highest risk of a pathological condition in the lower extremities. The efficiency of further initial triage before GPs can refer to CUS is questioned as over one fifth had DVT when referred by their GP. It seems reasonable to examine patients at this level of risk of DVT.

The fast, direct-access to CUS without pre-test and D-dimer for the initial assessment of patients suspected for DVT will be easy for the GP, optimal for the patient and need fewer resources. We primarily used a sonographer to perform CUS instead of a radiologist and a task shifting from radiologist to sonographer will require investments in education. However, it seems relevant and safe to use a sonographer and will provide more time for the radiologist to perform other tasks. Had we used a radiologist, the direct-access pathway still costed less (€14,520) than the previous pathway, equivalent to €32.3 less per patient (data not shown).

## Conclusion

In conclusion, implementation of the direct-access pathway for GPs for patients suspected for DVT resulted in faster pathways for the patients. Around one fourth of referred patients had a DVT and the two-way route gave GPs the possibility to indicate patients with high risk of DVT. The new, fast-track pathway appeared safe and needed fewer resources when compared to previous pathways.

## Data Availability

The study is based on a dataset not publicly available due to the restrictions imposed by the approval from the Danish Data Agency. However, the dataset are available from the corresponding author on reasonable request.

## Abbreviations

DVT: Deep vein thrombosis
GPs: General practitioners
CUS: Compression ultrasound
MOC: Medical Outpatient Clinic
RIS: Radiology Information System
IDDVT: Isolated distal deep vein thrombosis

## References

[1] Engbers MJ, van Hylckama VA, Rosendaal FR (2010). Venous thrombosis in the elderly: incidence, risk factors and risk groups. J Thromb Haemost.

[2] Naess IA, Christiansen SC, Romundstad P, Cannegieter SC, Rosendaal FR, Hammerstrom J (2007). Incidence and mortality of venous thrombosis: a population-based study. J Thromb Haemost.

[3] Mazzolai L, Aboyans V, Ageno W, Agnelli G, Alatri A, Bauersachs R (2018). Diagnosis and management of acute deep vein thrombosis: a joint consensus document from the European society of cardiology working groups of aorta and peripheral circulation and pulmonary circulation and right ventricular function. Eur Heart J..

[4] Bates SM, Jaeschke R, Stevens SM, Goodacre S, Wells PS, Stevenson MD (2012). Diagnosis of DVT: antithrombotic therapy and prevention of thrombosis, 9th ed: American College of Chest Physicians Evidence-Based Clinical Practice Guidelines. Chest.

[5] NICE (2012). Venous thrombolic diseases: the management of venous thrombophilia testing.

[6] Geersing GJ, Zuithoff NP, Kearon C, Anderson DR, Ten Cate-Hoek AJ, Elf JL (2014). Exclusion of deep vein thrombosis using the Wells rule in clinically important subgroups: individual patient data meta-analysis. BMJ.

[7] Mantoni MY, Kristensen M, Brogaard MH, Sivertsen JC, Nielsen JD, Strandberg C (2008). Diagnostic strategy in patients with clinically suspected deep vein thrombosis. Ugeskr Laeger.

[8] Kraaijenhagen RA, Piovella F, Bernardi E, Verlato F, Beckers EA, Koopman MM (2002). Simplification of the diagnostic management of suspected deep vein thrombosis. Arch Intern Med.

[9] Johnson SA, Stevens SM, Woller SC, Lake E, Donadini M, Cheng J (2010). Risk of deep vein thrombosis following a single negative whole-leg compression ultrasound: a systematic review and meta-analysis. JAMA.

[10] Horner D, Hogg K, Body R, Jones S, Nash MJ, Mackway-Jones K (2014). Single whole-leg compression ultrasound for exclusion of deep vein thrombosis in symptomatic ambulatory patients: a prospective observational cohort study. Br J Haematol.

[11] Gibson NS, Schellong SM, Kheir DY, Beyer-Westendorf J, Gallus AS, McRae S (2009). Safety and sensitivity of two ultrasound strategies in patients with clinically suspected deep venous thrombosis: a prospective management study. J Thromb Haemost.

[12] Pedersen CB, Gotzsche H, Moller JO, Mortensen PB (2006). The Danish Civil Registration System. a cohort of eight million persons. Dan Med Bull.

[13] Danmarks Nationalbank. Nationalbankens Statbank - Exchange rates. Available at: http://nationalbanken.statistikbank.dk/statbank5a/default.asp?w=1366. Accessed 16 Feb 2018.

[14] Ten Cate-Hoek AJ, Prins MH (2005). Management studies using a combination of D-dimer test result and clinical probability to rule out venous thromboembolism: a systematic review. J Thromb Haemost.

[15] Ageno W, Camporese G, Riva N, Iotti M, Bucherini E, Righini M (2015). Analysis of an algorithm incorporating limited and whole-leg assessment of the deep venous system in symptomatic outpatients with suspected deep-vein thrombosis (PALLADIO): a prospective, multicentre, cohort study. Lancet Haematol.

[16] Mumoli N, Vitale J, Giorgi-Pierfranceschi M, Sabatini S, Tulino R, Cei M (2017). General practitioner-performed compression ultrasonography for diagnosis of deep vein thrombosis of the leg: a multicenter, prospective cohort study. Ann Fam Med.

[17] Bernardi E, Camporese G, Buller HR, Siragusa S, Imberti D, Berchio A (2008). Serial 2-point ultrasonography plus D-dimer vs whole-leg color-coded Doppler ultrasonography for diagnosing suspected symptomatic deep vein thrombosis: a randomized controlled trial. JAMA.

[18] Ho P, Lim HY, Chua CC, Sleeman M, Tacey M, Donnan G (2016). Retrospective review on isolated distal deep vein thrombosis (IDDVT) - a benign entity or not?. Thromb Res.

[19] Porfidia A, Carnicelli A, Bonadia N, Pola R, Landolfi R (2016). Controversies in venous thromboembolism: the unique case of isolated distal deep vein thrombosis. Intern Emerg Med.

[20] Camporese G, Bernardi E, Scarano L, Ghirarduzzi A, Imberti D, Prandoni P (2012). Outcome of patients with suspected lower limb symptomatic deep vein thrombosis and a normal ultrasound-based initial diagnostic workup: a prospective study. J Thromb Haemost.

[21] Sutter ME, Turnipseed SD, Diercks DB, Samuel P, White RH (2009). Venous ultrasound testing for suspected thrombosis: incidence of significant non-thrombotic findings. J Emerg Med.

